# Leaf Fungal Endophyte Differs Among Plant Functional Groups in an Alpine Meadow

**DOI:** 10.1002/ece3.73239

**Published:** 2026-03-12

**Authors:** Miao Dong, Xiaogang Li, Xiaoli Hu, Shucun Sun

**Affiliations:** ^1^ Department of Biology, School of Life Sciences Nanjing University Nanjing China; ^2^ College of Biology and the Environment Nanjing Forestry University Nanjing China

**Keywords:** leaf fungal endophyte, leaf mass per area, nitrogen concentration, plant functional group, species abundance

## Abstract

Although numerous studies have documented the differences in leaf fungal endophyte (LFE) communities among various plant species inhabiting the same environments, the disparities in LFE among distinct species groups have rarely been examined at the community level. The composition and structure of the LFE community are known to be influenced by the abundance of host plants and the leaf functional traits at the species level. Given that various plant functional groups exhibit differences in relative abundance and leaf functional traits, we hypothesize that these distinct plant functional groups may support unique LFE communities, which are likely correlated with their specific functional demands. In this study, we investigated LFE community across 45 plant species, which were categorized into four functional groups: grasses, legumes, dicot forbs, and monocot forbs from an alpine meadow, utilizing high‐throughput sequencing techniques. We assessed the differences in LFE among the plant functional groups and analyzed these differences in relation to plant abundance and leaf functional traits. The LFE community exhibited significant differences among plant functional groups. The dicot forbs demonstrated a higher richness of LFE compared to the other three functional groups. Ascomycota was found to be the dominant phylum across all plant functional groups. Additionally, marker operational taxonomic units (OTUs) associated with a symbiotic lifestyle were more prevalent in legumes than in the other three functional groups. Leaf mass per area is identified as the primary determinant of variation in LFE community across different plant functional groups, with water content and leaf nitrogen concentration serving as secondary factors. Furthermore, species abundance also plays a significant role in explaining the variation observed in LFE. Our research enhances the understanding of microbial‐plant interactions and indicates a potential role of LFEs in shaping community structure and dynamics.

## Graphical abstract

The communities of leaf fungal endophyte had significant differences among plant functional groups and were affected by plant abundance and leaf functional traits among them in an alpine meadow.

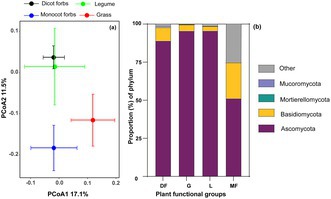

## 1 Introduction

Leaf fungal endophytes (LFEs) reside in the cells or intercellular spaces of plant leaves (Rodriguez et al. 2009). They can enhance plant resistance to pathogens and herbivores by producing antimicrobial metabolites and toxic substances, in exchange for resources from the host (Mousa et al. 2015; Zhang et al. 2009). Additionally, LFEs may improve plant tolerance to abiotic stress by regulating plant hormone production (Khan et al. 2011; Llorens et al. 2019). Thus, LFEs are functionally important for plant performance and may further influence plant survival and population growth.

Not all plant species host the same LFE communities. Consistent research has shown that LFE communities differ among plant species, even those growing in the same habitat (González‐Teuber et al. 2020; Currie et al. 2013; Yang et al. 2025). For example, in a temperate rainforest in southern Chile, LFE communities vary significantly among tree species, with this variation correlated to leaf resistance traits such as cell wall composition, flavonoid content, anthocyanins, and terpenoids (González‐Teuber et al. 2020). Similarly, the composition of leaf endophyte communities in herbaceous plants is influenced by both fungal and host factors, including plant genotype, defense mechanisms, and geographical location (Currie et al. 2013). These findings highlight the key role of plant functional traits in driving differences in endophyte communities.

Despite the importance of plant functional groups in regulating species performance and ecosystem functioning (Gitay and Noble 1997; Lavorel and Garnier 2002), few studies have investigated whether different plant functional groups are characterized by distinct LFE communities (Wang et al. 2017). Plant functional groups are widely used in community and ecosystem ecology (e.g., Hu et al. 2021; Liu et al. 2018; Ganjurjav et al. 2016). Establishing a link between LFE communities and plant functional groups could expand the utility of these classifications. Furthermore, given the role of LFEs in ecosystem primary productivity and nutrient cycling, understanding the relationship between plant functional groups and LFE communities is essential for predicting ecosystem responses to environmental changes, such as climate change and land‐use modification.

LFE communities may be associated with plant functional type because different plant functional groups have distinct leaf functional traits (LFTs) (Tsakalos et al. 2019), and these traits regulate endophyte entry, colonization, and community composition (González‐Teuber et al. 2021). Indeed, LFE communities are known to be strongly affected by LFTs such as leaf mass per area (LMA), water content, nutrient concentrations, and carbon‐to‐nitrogen (C:N) ratio (Vincent et al. 2016; Liu et al. 2019; Tellez 2019; Oono et al. 2020; Li et al. 2020). For instance, variation in LFE community composition is partially explained by the LMA, nitrogen and carbon content across tree species in New Guinea rainforests (Vincent et al. 2016). Likewise, in the neotropical rainforest in Panama, the LFE abundance and species diversity of 30 tree species are negatively associated with LMA but positively associated with foliar nitrogen (Tellez 2019). Additionally, LFE abundance is linked to the C:N ratio in *Pinus muricata* and *Vaccinium ovatum* (Oono et al. 2020). High leaf water content significantly increases the infection rate of endophytic fungi in *Achnatherum inebrians* (Li et al. 2020), while water deficiency reduces the frequency of endophytic fungi in leaves of *Cenangium ferruginosum* (Lee et al. 2014). Water content also explains a large portion of variation in endophytic fungal community composition in *Cirsium arvense* (Eschen et al. 2010). Thus, we hypothesize that plant groups with lower LMA, lower C:N ratio, and higher water content will have higher LFE abundance and diversity.

To test this hypothesis, we investigated LFEs across 45 plant species (belonging to 18 families) in an alpine meadow on the Tibetan Plateau. These species were classified into four functional groups: grasses, legumes, dicot forbs, and monocot forbs. The objectives of this study were to: (1) determine whether LFE composition varies among different plant functional groups; (2) examine whether host functional traits explain observed variation in LFE communities among these groups; and (3) assess whether species abundance contributes significantly to LFE variation among plant functional groups (based on the “common host hypothesis,” which proposes that sufficiently abundant plant populations enable passively dispersed endophytes to reliably recolonize the same species; Apigo and Oono 2022).

## 2 Materials and Methods

### 2.1 Study Site

This study was conducted in an alpine meadow in Hongyuan County, Sichuan Province, Southwest China, located on the eastern Tibetan Plateau (32°83′ N, 102°58′ E). The site has an elevation of approximately 3500 m above sea level. The mean annual temperature is around 1.7°C, with the minimum monthly mean temperature (−9.3°C) in January and the maximum (11.1°C) in July. Annual mean precipitation is 756 mm, but it fluctuates annually (ranging from 450 to 900 mm). Over 80% of annual precipitation occurs between May and September.

The alpine meadow is primarily grazed by yaks ( *Bos grunniens* ) during the nongrowing season. The ecosystem has high plant diversity, with approximately 25–30 species per 1 m^2^. Vegetation coverage exceeds 90%, including various forbs (e.g., *Saussurea nigrescens*, *Anaphalis flavescens*, *Potentilla discolor*, *Polygonum viviparum* ), grasses (e.g., *Deschampsia cespitosa* , *Festuca rubra* , *Elymus nutans*), and legumes (e.g., *Lathyrus quinquenervius*, *Hedysarum sikkimense*) (Li et al. 2011).

### 2.2 Leaf Trait and Species Abundance

In July 2020 (when leaves were mature), we collected leaf samples from five individuals for each of the 45 plant species. These species were assigned to four functional groups: grasses (G, six species), legumes (L, four species), dicot forbs (DF, 32 species), and monocot forbs (MF, three species) (Table S2).

Fresh leaf area was scanned using a Canon Lide 300 scanner (USA) and calculated with ImageJ software (version 1.53c; National Institutes of Health, Bethesda, MD, USA). Leaves were then dried for 48 h at 75°C and weighed. Carbon (C) and nitrogen (N) contents of dried leaves were determined using an Elementar Vario ELIII elemental analyzer (Germany). LMA was calculated as dried leaf weight divided by leaf area. Leaf water content (WC) was calculated as the percentage of fresh weight lost after drying [(fresh weight—dry weight)/fresh weight × 100%].

Plant community abundance and composition were surveyed using quadrat sampling, following Pauli et al. (2015). Twenty 0.5 × 0.5 m quadrats were randomly placed within a 100 × 150 m grassland area. Each quadrat was subdivided into 10 × 10 cm grids, and species presence and abundance were recorded following Hu et al. (2021).

### 2.3 Leaf Fungal Endophyte

Three replicates were conducted to analyze the total genomic DNA of the microbes. Each replicate consisted of approximately 0.8 g of fresh leaves, which were collected from five individuals (different from those sampled for leaf trait measurement) of each species. For each replicate, the leaf samples underwent surface sterilization using 75% ethanol for 2 min, followed by immersion in 0.5% sodium hypochlorite (NaClO) for 10 min. Subsequently, the samples were rinsed three times with sterile water. Ten sterilized leaves were randomly chosen to grow on PDA and no fungal growth was found. Total genomic DNA was extracted using Tiangen Plant DNA Kit (TIANGEN Biotech, Beijing, China).

The polymerase chain reaction (PCR) was performed following the extraction of DNA. Fungal primers, specifically ITS4‐Fun (5′‐AGCCTCCGCTTATTGATATGCTTAART‐3′) and gITS7F (5′‐GTGARTCATCGARTCTTTG‐3′), were selected to amplify the ribosomal DNA (rDNA) encompassing the internal transcribed spacer regions. All primers were synthesized by Biobert Biotechnologies Inc. (Chengdu, China). The reactions were carried out in a 25 μL mixture, which comprised 12.5 μL of 2× Taq MasterMix (TsingKe Biological Technology Co. Ltd., Beijing, China), 1 μL of forward primer, 1 μL of reverse primer, 9.5 μL of double‐distilled water (ddH2O), and 1 μL of DNA at a concentration of 10 ng/μL. The PCR amplification protocol included an initial denaturation step at 94°C for 5 min, followed by 34 cycles of denaturation at 94°C for 30 s, 56°C for 30 s, and 68°C for 45 s, concluded with a final extension at 72°C for 10 min.

Libraries were constructed using the TruSeq DNA PCR‐Free Sample Preparation Kit (Illumina, San Diego, CA, USA), and amplicon sequencing was conducted using the paired‐end 2 × 250 bp Illumina MiSeq Benchtop Sequencer (Illumina, San Diego, CA, USA) at Biobert Biotechnologies Inc. (Chengdu, China). Low‐quality reads (length < 200 bp, more than two ambiguous bases ‘N' and average base quality score < 30) were filtered. After finding duplicated sequences, we discarded all the singletons (http://www.drive5.com/usearch/manual/singletons.html). Sequences were clustered into operational taxonomic units (OTUs) at a 97% similarity threshold using UPARSE algorithms. Potential chimeras in representative sequences were removed by the Uchime algorithm. Taxonomic assignment was conducted using the UNITE database (version 8.0) as a reference with ≥ 97% similarity threshold and representative sequences were aligned using PyNAST embedded in QIIME v1.9.1. Functional information for OTUs was assigned using FUNGuild (Nguyen et al. 2021).

### 2.4 The Marker OTUs of LFE

We employed specificity and occupancy metrics for each OTU to filter marker OTUs within each plant functional group. As described by Dufrêne and Legendre (1997), specificity is defined as the mean abundance of an OTU across samples belonging to a particular plant functional group, while occupancy refers to the relative frequency of occurrence of the OTU within those samples. The calculations for specificity and occupancy were conducted as follows:

$$\text{Specificity}=\frac{{\text{Nindividuals}}_{S,H}}{{\text{Nindividuals}}_{S}}$$

$$\text{Occupancy}=\frac{{\text{Nsites}}_{S,H}}{{\text{Nsites}}_{S}}$$

Nindividual_*S*,*H*_ represents the mean number of individual OTUs across all samples within a given plant functional group, whereas Nindividual_S_ denotes the sum of the mean number of individual OTUs across all plant functional groups. Nsites_*S*,*H*_ refers to the number of samples within a plant functional group in which a given OTU is present, while Nsites_H_ indicates the total number of samples in a specific plant functional group. According to the methodology outlined by Gweon et al. (2021), OTUs with specificity and occupancy values ≥ 0.7 were selected as marker OTUs for each plant functional group. Identifying marker OTUs for fungal communities is crucial for breaking down complex DNA data into manageable, identifiable groups, allowing scientists to study fungal diversity, ecology, biogeography, and interactions with plants.

### 2.5 Statistical Analysis

The OTU richness and Shannon diversity indices were calculated utilizing the ‘diversity’ function from the R package vegan v. 2.5–4 (Oksanen et al. 2014). Generalized linear models (GLMs) were employed to assess the differences in alpha diversity of LFE among different plant functional groups, using the ‘glm’ function within the same R package. The beta diversity of LFE based on Bray–Curtis distance, was analyzed through Principal Coordinates Analysis (PCoA). Additionally, the differences in endophyte community composition among the distinct plant functional groups were evaluated using pairwise permutational multivariate analysis of variance (PERMANOVA), implemented via the ‘pairwise.adonis2’ function in the R package pairwiseAdonis v. 0.4 (Martinez 2017). The phylogenetic tree of plant was inferred from mega tree of Open Tree of Life, using ‘get_tree’ function in the R package rtrees v. 1.0.3 (Li 2023). Pairwise phylogenetic distances among plant species were calculated by the phylogenetic tree, using ‘cophenetic’ function in the R package phangorn v. 2.11.1 (Schliep 2011). In addition, the Bray–Curtis distances of the LFE community, the Euclidean distances of each leaf functional trait and plant abundance among species from different functional groups, were calculated using ‘vegdist’ function in the R package vegan v. 2.5–4.

A multivariate linear mixed model (LMM) was constructed to determine the effects of plant phylogeny, leaf function traits, and plant abundance (reflected by phylogenetic distances, Euclidean distances of LFTs and plant abundance between each species pair across plant functional groups) on LFE community composition (reflected by the Bray–Curtis distances of the LFE community). The identity of plant species was included as a random factor in the model (see Rodrigues et al. 2024). The ‘glmm.hp’ function from the R package glmm.hp. version 0.1–5 was applied to quantify the explanatory ratios of predictor variables for the variance of LFE community among plant functional groups (Lai et al. 2022, 2023).

The variation in the functions of marker OTUs composition was assessed using PCoA. The significance of the variation among each plant functional group was evaluated through pairwise permutational multivariate analysis of variance (PERMANOVA), employing the ‘pairwise.adonis2’ function from the R package pairwiseAdonis version 0.4 (Martinez 2017).

## 3 Results

### 3.1 Difference in Fungi Community Compositions Among Plant Functional Groups

**FIGURE 1:**
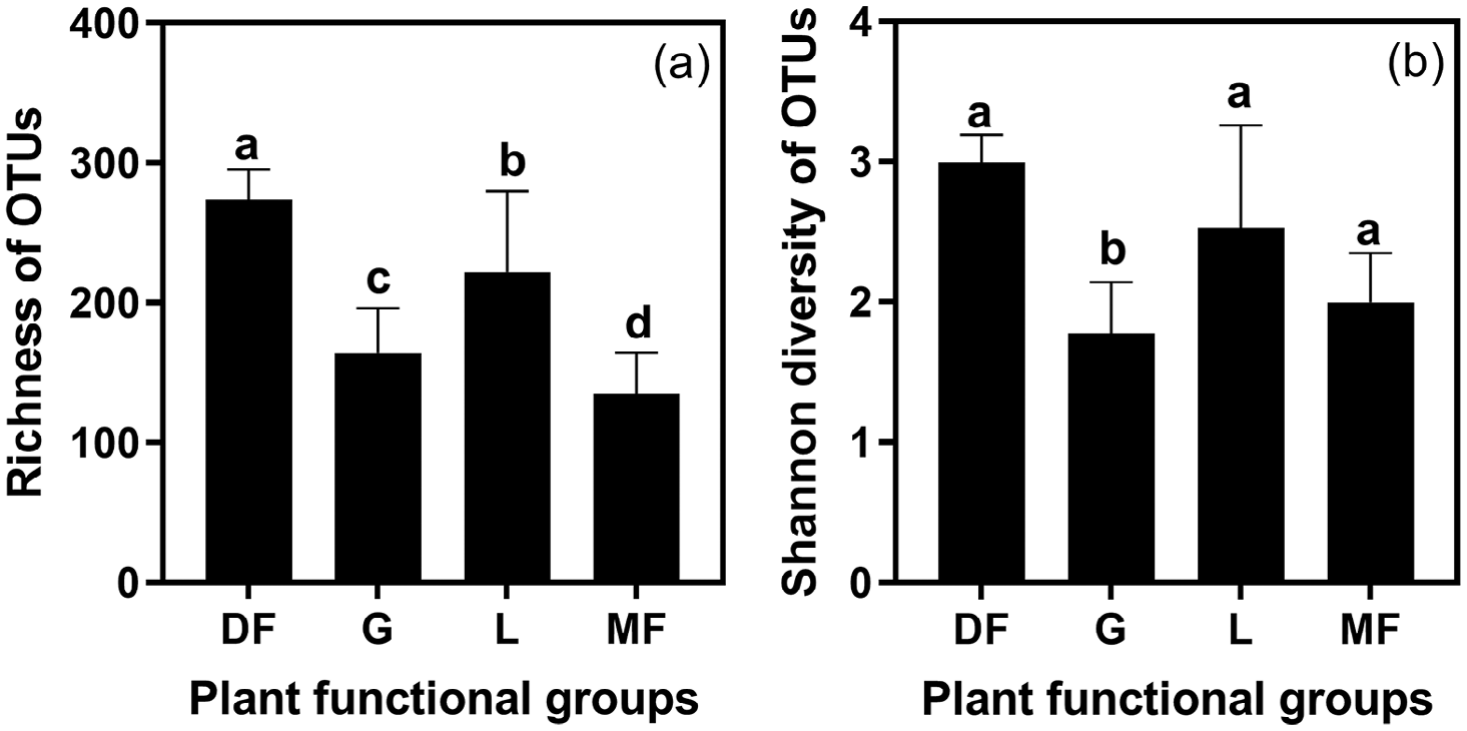
Difference in OTU richness (a) and shannon diversity (b) of LFE communities among the four plant functional groups. Different letters above the bars denote statistically significant differences among plant functional groups at the level of *p* < 0.05.

A total of 196,162 high‐quality sequences were obtained, which clustered into 2273 OTUs for endophytic fungi. Among these, legumes comprised 580 OTUs, grasses 505 OTUs, dicot forbs 2153 OTUs, and monocot forbs 312 OTUs. The average OTU richness of LFE differed significantly among all plant functional groups, with values of 273.4 for dicot forbs, 163.7 for grasses, 221.8 for legumes and 134.3 for monocot forbs (see Figure 1a). Additionally, the Shannon diversity of LFE in grasses was significantly lower than that of the other three plant functional groups (see Figure 1b). The richness of OTUs was significantly and positively influenced by plant abundance, nitrogen concentration, and water content within the plant functional groups, while it was significantly and negatively influenced by the LMA of the plant functional groups (see Table 1). Furthermore, the Shannon diversity of OTUs was significantly and positively influenced by the abundance of the plant functional groups (see Table 1).

**TABLE 1:** Results of linear models showing the factors affecting richness and Shannon diversity of OTU among plant functional groups. Multivariate analysis was not conducted due to the collinearity among different factors in relation to OTU richness and diversity. *N* = 45 for each model.

*Y*	*X*	Estimate	*p*
OTU richness	Species abundance	0.1001	< 0.001
	Leaf nitrogen concentration	0.0376	0.0738
	Leaf C: N	0.0058	0.2
	Leaf water content	1.9519	< 0.001
	Leaf mass per area	−83.5919	< 0.001
OTU diversity	Species abundance	0.1965	0.018
	Leaf nitrogen concentration	0.1565	0.698
	Leaf C: N	−0.0016	0.984
	Leaf water content	6.5775	0.147
	Leaf mass per area	−225.0977	0.076

**FIGURE 2:**
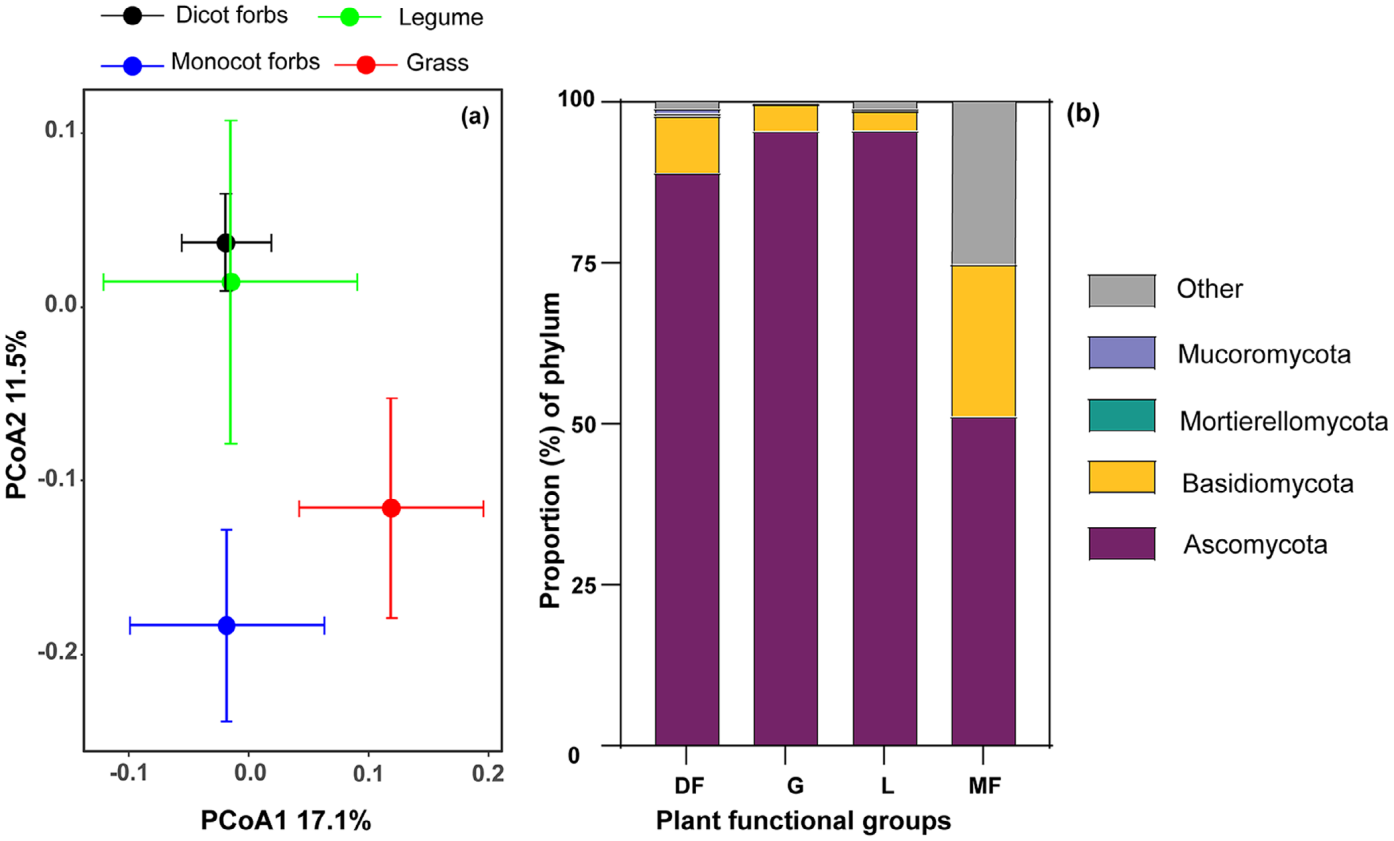
PCoA plot of the LFE composition (a) based on bray‐curtis distances at the OTU level, colored by plant functional groups. Centroids and standard errors are used to represent points in each plant functional group; relative abundance of LFE (b) in all plant functional groups at phylum level.

Pairwise PERMANOVA analyses indicated that the LFE compositions exhibited significant differences between dicot forbs and grasses, as well as between dicot forbs and monocot forbs. However, no significant differences were detected among the remaining pairwise combinations of plant functional groups (see Figure 2a; Table S1). Ascomycota were the predominant phylum across all plant functional groups, comprising 89.1% of the community in dicot forbs, 95.6% in grasses, 95.8% in legumes, and 51.2% in monocot forbs. Within dicot forbs, Basidiomycota constituted 8.8%, Mucoromycota approximately 0.7%, and Mortierellomycota around 0.4%. Approximately 0.9% of the LFE in dicot forbs were classified as unidentified or too sparse to be categorized at the phylum level, and were thus grouped under ‘Other’. In grasses and legumes, Basidiomycota represented 4.2% and 2.9%, respectively, while other phyla were present in minor proportions. In monocot forbs, Basidiomycota accounted for 23.6%, and the ‘Other’ category comprised 25.2% (Figure 2b).

### 3.2 Factors Affecting LFE Community Among Plant Functional Groups

The variation in LMA emerged as the most significant factor influencing changes in the composition of LFE community across different plant functional groups. Additionally, the variation in plant abundance exerted a greater impact on the compositional changes within the LFE community than the other examined plant functional traits. Furthermore, differences in water content and nitrogen concentration also contributed to variation in the composition of LFE communities among the various plant functional groups. In contrast, the variation in the C:N and plant phylogeny demonstrated the weakest and statistically insignificant effects on the compositional changes within the LFE communities across the plant functional groups (Table 2).

**TABLE 2:** Results of multivariate linear mixed model showing the effect of species abundance and functional traits on LFE community composition among plant functional groups. *N* = 470.

*Y*	*X*	*F*	*p*	Individual importance (*R* ^2^)
LFE community composition	Species abundance	14.5465	< 0.001	0.027
	Leaf nitrogen concentration	9.3754	0.002	0.015
	Leaf C: N	0.3278	0.567	0.004
	Leaf water content	5.8704	0.015	0.017
	Leaf mass per area	13.1701	< 0.001	0.037
	Plant phylogeny	1.5313	0.216	0.004

### 3.3 Difference in Marker OTUs Among Plant Functional Groups

**FIGURE 3:**
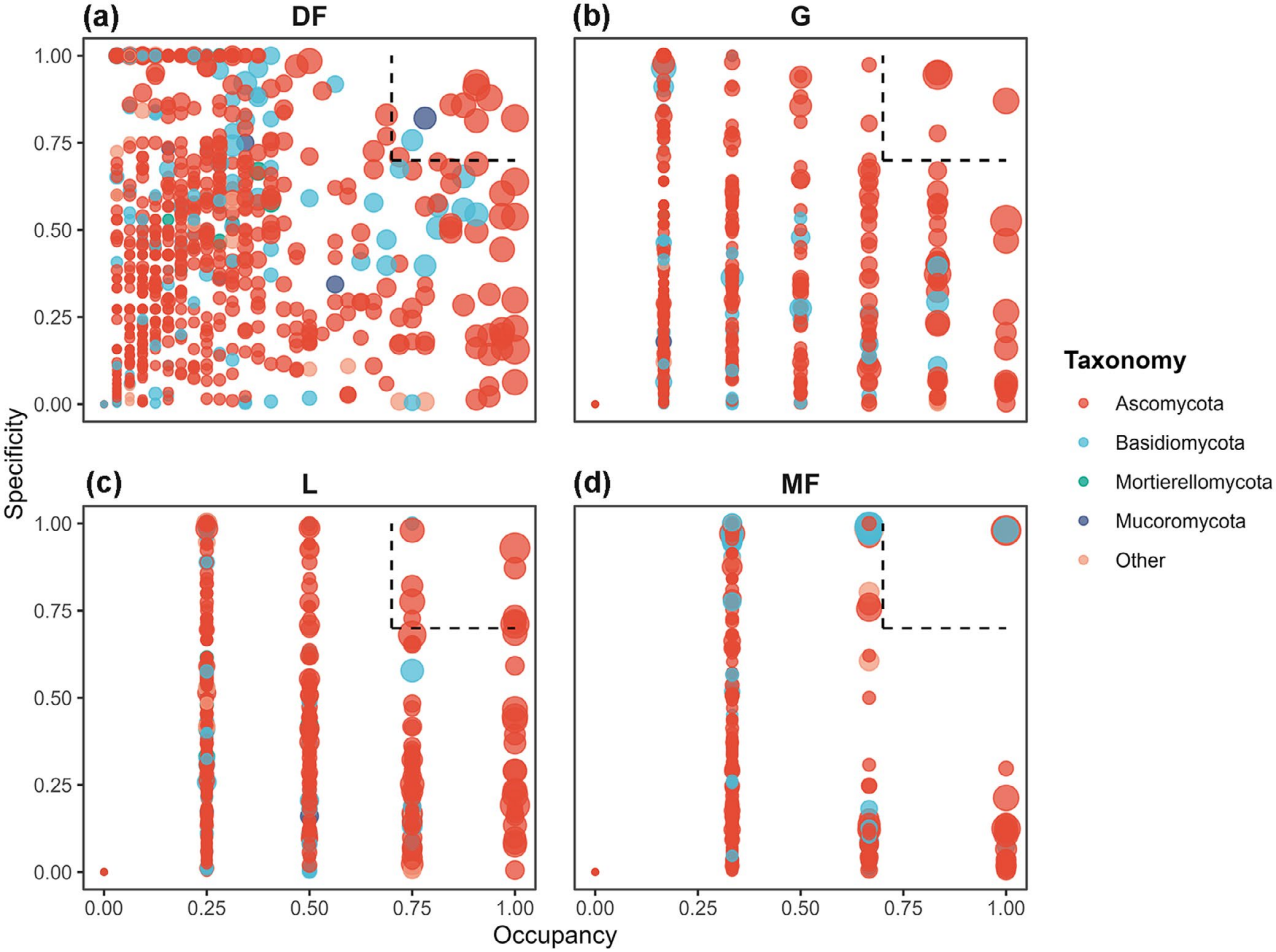
The SPEC‐OCCU plots show marker OTUs in each plant functional group. The *x*‐axis represents occupancy and the *y*‐axis represents specificity. The points within the dashed box are the marker OTUs. (a) Dicot forbs; (b) Grasses; (c) Legumes; and (d) Monocot forbs.

LFEs within all plant functional groups demonstrated a considerable variation in occupancy and specificity values. Based on these values, we identified 27 marker OTUs distributed among the four plant functional groups. The quantity of these marker OTUs differed among the groups, revealing an increasing trend in richness: monocot forbs (three marker OTUs), grass (four marker OTUs), dicot forbs (10 marker OTUs), and legume (10 marker OTUs). These groups accounted for 21.5%, 62.6%, 2.7%, and 16.6% of the total sequences, respectively. Ascomycota were identified in the marker OTUs for all four plant functional groups. Notably, the marker OTUs associated with dicot forbs included representatives from Basidiomycota and Mucoromycota, while those in monocot forbs also encompassed Basidiomycota (Figure 3).

**FIGURE 4:**
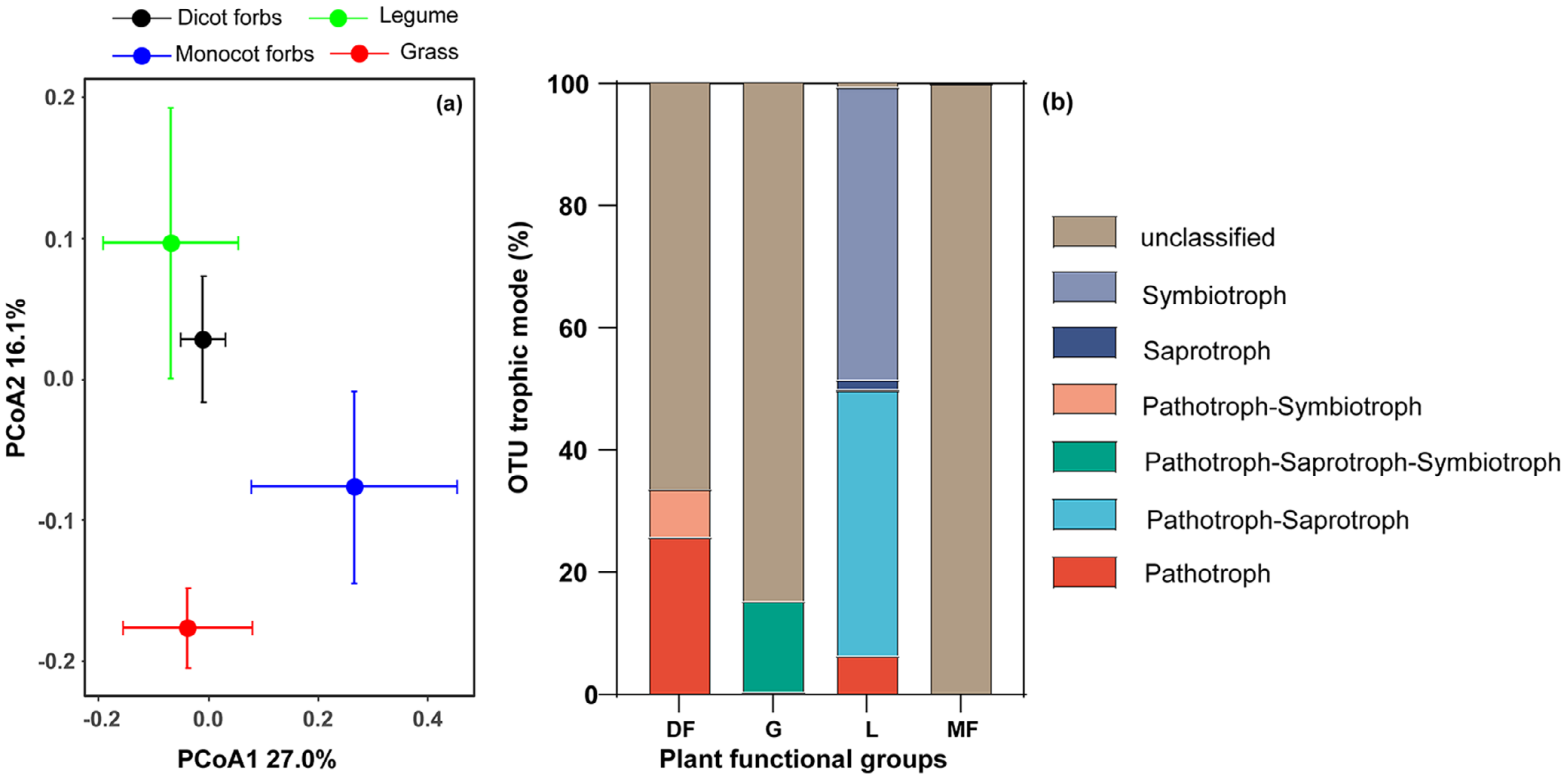
PCoA plot of the functional composition (a) of marker OTUs based on bray‐curtis distances at functional level, colored by plant functional groups. Centroids and standard errors are used to represent points in each plant functional group; (b) Relative abundance of functional marker OTUs of LFE among plant functional groups.

Pairwise PERMANOVA analyses indicated that the functional composition of marker OTUs among plant functional groups was significantly different between dicot forbs and legumes, dicot forbs and monocot forbs, grasses and legumes, as well as legumes and monocot forbs. However, no significant differences were detected between dicot forbs and grasses, nor between grasses and monocot forbs (see Figure 4a; Table S1). Furthermore, the symbiotic trophic mode of marker OTUs in legumes was found to be higher than that of the other three plant functional groups, while the pathogenic trophic mode of marker OTUs in dicot forbs was also higher than that of the other three groups (Figure 4b).

## 4 Discussion

We have shown that the variation in LFE community among different plant functional groups can be explainned by species abundance and functional traits including LMA, nitrogen concentration and water content but is not influenced by plant phylogeny, thereby supporting the initial hypothesis. Notably, the LFE community was indistinguishable between monocot forbs and grass species groups; however, it displayed differences between monocot forbs and dicot forbs. This finding expands the classification of plant functional groups, which typically categorizes monocot forbs and dicot forbs within the same functional group. Consequently, our study suggests that the LFE community may serve as a reliable indicator for differentiating plant functional groups, despite the observed inconsistencies.

### 4.1 Difference in LEF Community Composition Among Plant Functional Groups

The operational taxonomic unit (OTU) richness of LEF community exhibits significant variation among different plant functional groups in the present study. This variation is likely attributable to the influence of the assembly of horizontally transmitted LFE among plants, a process that is modulated by plant defense traits and resource traits, including LMA and nitrogen concentration. Previous research has indicated that endophyte colonization and dissemination are more effective in plants characterized by high carbon assimilation, low LMA, and high nutrient concentrations (Reich 2014; González‐Teuber et al. 2021). In alignment with these findings, dicotyledonous forbs and legumes exhibiting lower LMA demonstrated higher richness and diversity of leaf endophytes compared to other plant functional groups (Figure S1, Figure 1).

Ascomycota predominated in the LFE community across all plant functional groups. The LFEs originate from environmental sources and filtered from non‐rhizosphere soils, rhizoplane roots, the endosphere, the phylloplane, and the leaf endosphere (Yao et al. 2019). Previous studies have indicated that Ascomycota comprise nearly 80% of the fungal community in the soils of Tibetan alpine grasslands (Kang et al. 2022). This substantial presence of Ascomycota in the soil increases the likelihood of their entry into plants, thereby contributing to their dominance across all plant functional groups. Our findings align with research on fungal endophytes in forest and desert ecosystems, where Ascomycota are also identified as the dominant group (Arnold and Lutzoni 2007; Zuo et al. 2021). However, Basidiomycota represent a relatively higher proportion within the monocot forbs group compared to other plant functional groups, as supported by additional studies (Varanda et al. 2016; Nguyen et al. 2021). The leaves of monocot forbs exhibit the highest water content among the four plant functional groups, creating an optimal microenvironment for Basidiomycota, which require higher humidity levels (Boddy 1983). Furthermore, monocot forbs typically possess extended growth periods or are classified as perennial plants, providing slow‐growing Basidiomycota (Rungjindamai and Jones 2024) with a longer timeframe to complete their infection and dissemination processes.

The composition of the LFE community exhibits significant variation among different plant functional groups. Notably, legumes demonstrate a similar LFE composition to dicotyledonous forbs, while monocotyledonous forbs share similarities with grasses in their composition. This variation can be attributed to several factors, including functional traits and species abundance. LMA emerges as the most significant driver of variance in LFE community compositions across plant functional groups, corroborating previous research that indicates a strong correlation between LMA and variance in LFE communities among tree species (Kembel and Mueller 2014; Liu et al. 2019). LMA serves as an indicator of leaf thickness and tissue density, which are closely associated with leaf growth strategies. Plants characterized by low LMA typically possess thin leaves, loose tissues, and weaker physical barriers, and are often fast‐growing species with minimal investment in defense mechanisms. This trait facilitates more effective colonization and dissemination of LFE (González‐Teuber et al. 2021). Furthermore, LMA exhibits considerable variation among plant functional groups, with dicotyledonous forbs displaying a lower LMA compared to grasses, while being comparable to legumes and monocotyledonous forbs. Water content also plays a crucial role in influencing LFE community composition. Water is essential for spore germination and mycelial growth; higher water content promotes the expansion of cellular spaces and softens cell walls, thereby facilitating mechanical penetration by LFE. Additionally, water content accounts for variance in LFE community composition within tree species (Sanchez‐Azofeifa et al. 2012; Tellez et al. 2020). Nitrogen (N) is another critical resource for fungal growth (Gao et al. 2007; Wan et al. 2015), and it is plausible that nitrogen availability may have influenced the relative abundance of Ascomycota within the LFE community composition.

In addition to plant functional traits, plant abundance significantly influences the variation in LFE community composition. As the abundance of host plants increases, LFEs that are highly adapted to these hosts are more likely to establish and proliferate due to increased frequency of contact (Bever et al. 1997; Mangan et al. 2010). Bagchi et al. (2010) has noted that pathogens typically exhibit increased abundance as their host plants become more prevalent. The rise in plant abundance can lead to heightened intraspecific competition and a subsequent reduction in the resources that plants allocate to defense mechanisms, thereby facilitating the colonization of endophytic fungi (Karban et al. 1989). Furthermore, significant differences in plant abundance between dicot and monocot forbs may account for the observed disparities in LFE community composition between these two groups.

### 4.2 Difference in Marked OTUs Composition Among Plant Functional Groups

Marked OTUs exhibit a high degree of specificity to particular plant functional groups while also being prevalent across all sites within those groups (Dufrêne and Legendre 1997). The composition of marker OTUs demonstrated significant differences among various plant functional groups, which is indicative of the disparities in the relative abundances of marker OTUs associated with pathogenic, symbiotic, and saprotrophic trophic modes. Similar to LFE community, plant functional traits and species abundance are significantly and partially responsible for the observed variation in marked OTU composition among plant functional groups. This finding suggests that, in addition to species functional traits, distinct plant functional groups are also correlated with different functional LFE communities.

The proportion of OTUs exhibiting a symbiotic lifestyle is significantly higher in legumes compared to the other three plant functional groups. This finding is consistent with previous studies that have demonstrated the symbiotic relationships between legumes and microorganisms (Dudeja et al. 2012). The OTUs associated with a symbiotic lifestyle predominantly include *Cadophora finlandica*. While *Cadophora finlandica* has been primarily reported as a root‐colonizing fungus (Dučić et al. 2009), our results revealed that it was abundantly present in the leaves of legumes, which may enhance plant growth by improving the nitrogen useing efficiency of their host (Alberton et al. 2010). Furthermore, legumes typically possess higher leaf nitrogen concentration, which may render them more susceptible to herbivory stress (Lan et al. 2021). The increased proportion of symbiotic LFEs may bolster the resistance of legumes to herbivores in alpine meadow ecosystems. These findings suggest that legumes exhibit a greater reliance on LFEs, particularly symbiotic LFEs, compared to the other three plant functional groups.

The dicot forbs exhibit a significantly higher proportion of marker OTUs with pathogenic trophic modes compared to the other three plant functional groups. This phenomenon can be attributed to its LMA, which creates more favorable conditions for the colonization and transmission of fungal pathogens (González‐Teuber et al. 2021). Furthermore, the dicot forbs demonstrate a greater relative species abundance, which is frequently positively correlated with the presence of pathogenic fungi (Liang et al. 2016). It is important to note that, although marker OTUs exhibiting either a strictly pathogenic or potentially pathogenic trophic mode are present in dicot forbs, grasses, and legumes, the host plants do not display overt signs of disease. This may be due to the hypothesis that the latent LFE of plants are thought to have evolved from pathogenic fungi and are closely related to virulent pathogens, yet exhibit limited pathogenic effects through extended latency periods (Carroll 1988; Petrini et al. 1992).

The marker OTUs with triple trophic modes in grass predominantly consist of *Aureobasidium pullulans*. Previous research has demonstrated that *Aureobasidium pullulans* can reduce plant microbial diversity by exhibiting rapid growth and competing for both nutrients and space, thereby creating an environment that is less favorable or even hostile to other microbial species (Bozoudi and Tsaltas 2018). In the monocot forbs, all three marker OTUs are classified as having an unclassified lifestyle, as they are only annotated to the phylum level in the sequencing database. This observation suggests that the functional ecology of the monocot forbs warrants further investigation through alternative research methodologies, such as morphological identification and metagenomics.

## 5 Conclusion

Our study shows that LFE community exhibits variability among different plant functional groups within alpine meadows. This finding indicates that plant functional groups, which are typically classified based on species traits, life histories, or responses to environmental changes, are also distinguished by their respective LFE communities. Furthermore, as different LFEs or OTUs possess distinct functional adaptive significance for plants, this diversity enables plants to respond differentially to environmental changes (Jia et al. 2020). Notably, our research reveals that both LFTs and species abundance can partially explain the variation observed in the LFE community, thereby reinforcing the correlation between plant functional groups and LFE communities. However, it is important to note that plant functional groups do not consistently align with differences in LFE. Specifically, the LFE of monocot forbs is comparable to that of grasses, yet distinct from that of dicot forbs, suggesting that the LFE of monocot forbs is more closely related to plant phylogeny than to the current classification of plant functional groups. Similarly, although legumes and dicot forbs are frequently categorized into separate plant functional groups, their LFE communities exhibit similarities in OTU composition. Consequently, given that monocot forbs are phylogenetically akin to dicot forbs while also exhibiting leaf traits similar to those of dicot forbs, we propose that the classification of monocot forbs within the dicot forbs functional group warrants further consideration and more species belonging to monocot forbs need to be included in the future research.

## Author Contributions

**Miao Dong:** data curation (lead), formal analysis (lead), investigation (lead), writing – original draft (lead), writing – review and editing (equal). **Xiaogang Li:** formal analysis (supporting). **Xiaoli Hu:** formal analysis (supporting). **Shucun Sun:** funding acquisition (lead), methodology (equal), supervision (lead), writing – review and editing (equal).

## Conflicts of Interest

The authors declare no conflicts of interest.

## Supporting information


**Data S1:** ece373239‐sup‐0001‐DataS1.docx.

## Data Availability

Source data will be archived on Zenodo by https://doi.org/10.5281/zenodo.18042478.
