# Supplementary material for: Leaf Fungal Endophyte Differs Among Plant Functional Groups in an Alpine Meadow
Source: Ecol Evol. 2026 Mar 12;16(3):e73239. doi: 10.1002/ece3.73239 (PMC13093845; doi:10.1002/ece3.73239)
Supplement: Supplementary file 1 — Data S1: ece373239‐sup‐0001‐DataS1.docx. [file ECE3-16-e73239-s001.docx]

**Supplementary information**

Table S1 Results of pairwise PERMANOVA showing the difference of LFE community composition and marked OTUs’ function among plant functional groups

Table S2 The functional group of each plant species

Figure S1 Leaf functional traits and plant abundance of plant functional groups. Different letters above the bars denote statistically significant differences among plant functional groups at the level of p < 0.05.

Table S1

|  |  | F | | p |
| --- | --- | --- | --- | --- |
| LFE community composition | whole model | | 1.6332 | 0.005 |
|  | DF vs G | | 2.2885 | 0.007 |
|  | DF vs L | | 1.0927 | 0.277 |
|  | DF vs MF | | 1.6505 | 0.049 |
|  | G vs L | | 1.5558 | 0.079 |
|  | G vs MF | | 1.3704 | 0.117 |
|  | L vs MF | | 1.2784 | 0.17 |
| Functional composition of marked OTUs | whole model | 2.6402 | | 0.006 |
|  | DF vs G | 0.7622 | | 0.537 |
|  | DF vs L | 3.4673 | | 0.026 |
|  | DF vs MF | 2.9671 | | 0.041 |
|  | G vs L | 3.0989 | | 0.018 |
|  | G vs MF | 2.6953 | | 0.145 |
|  | L vs MF | 6.5084 | | 0.032 |

Table S2

| Plant species | Plant functional groups |
| --- | --- |
| *Gentiana abaensis* | Dicot forbs |
| *Trollius farreri* | Dicot forbs |
| *Anemone rivularis* | Dicot forbs |
| *Taraxacum lugubre* | Dicot forbs |
| *Elymus nutans* | Grasses |
| *Koeleria macrantha* | Grasses |
| *Tongoloa elata* | Dicot forbs |
| *Anaphalis flavescens* | Dicot forbs |
| *Angelica sinensis* | Dicot forbs |
| *Saussurea nigrescens* | Dicot forbs |
| [*Argentina anserina*](https://www.iplant.cn/info/Argentina%20anserina) | Dicot forbs |
| *Sibbaldianthe bifurca* | Dicot forbs |
| *Deschampsia cespitosa* | Grasses |
| *Potentilla discolor* | Dicot forbs |
| *Tibetia himalaica* | Legumes |
| *Allium sikkimense* | Monocot forbs |
| *Aster alpinus* | Dicot forbs |
| *Oxytropis ochrocephala* | Legumes |
| *Ligularia virgaurea* | Dicot forbs |
| *Leontopodium leontopodioides* | Dicot forbs |
| *Agrostis hugoniana* | Grasses |
| *Galium spurium* | Dicot forbs |
| *Delphinium caeruleum* | Dicot forbs |
| *Geranium wilfordii* | Dicot forbs |
| *Artemisia frigida* | Dicot forbs |
| *Scutellaria hypericifolia* | Dicot forbs |
| *Veronica eriogyne* | Dicot forbs |
| *Ajuga ovalifolia* | Dicot forbs |
| *Gentiana formosa* | Dicot forbs |
| *Euphorbia esula* | Dicot forbs |
| *Lathyrus quinquenervius* | Legumes |
| *Thalictrum pseudoramosum* | Dicot forbs |
| *Anemone coelestina* | Dicot forbs |
| *Halenia elliptica* | Dicot forbs |
| *Hedysarum sikkimense* | Legumes |
| *Ajania przewalskii* | Dicot forbs |
| *Poaceae Poa* | Grasses |
| *Euphrasia pectinata* | Dicot forbs |
| *Saussurea stella* | Dicot forbs |
| *Pedicularis anas* | Dicot forbs |
| *Festuca ovina* | Grasses |
| *Allium chrysanthum* | Monocot forbs |
| *Silene gallica* | Dicot forbs |
| *Iris tectorum* | Monocot forbs |
| *Aster diplostephioides* | Dicot forbs |


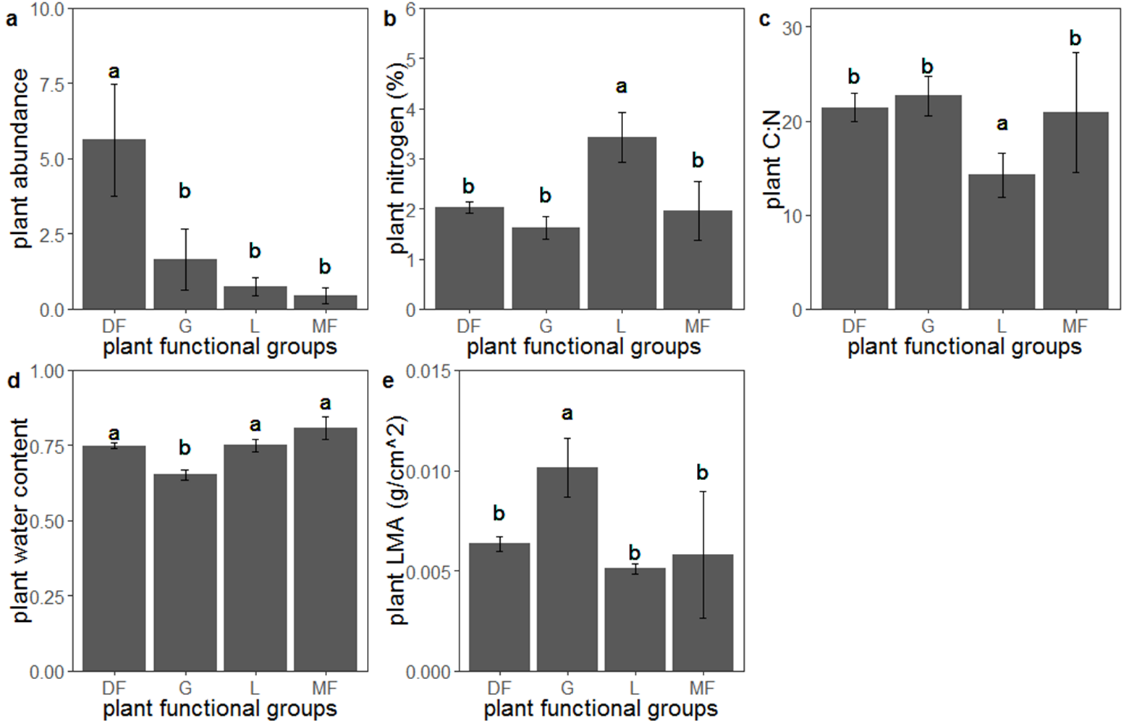


Figure S1
